# High interleukin-8 level in aqueous humor is associated with poor prognosis in eyes with open angle glaucoma and neovascular glaucoma

**DOI:** 10.1038/s41598-018-32725-3

**Published:** 2018-09-28

**Authors:** Ikuyo Chono, Dai Miyazaki, Hitomi Miyake, Naoki Komatsu, Fumie Ehara, Daisuke Nagase, Yukimi Kawamoto, Yumiko Shimizu, Ryuichi Ideta, Yoshitsugu Inoue

**Affiliations:** 1 Tottori University, Division of Ophthalmology and Visual Science, Faculty of Medicine, 36-1 Nishi-cho, Yonago, Tottori, 683-8504 Japan; 2 Ideta Eye Hospital, 39 Nishitojinmachi Chuo-ku Kumamoto, Kumamoto, 860-0027 Japan

## Abstract

Glaucoma is a leading cause of blindness worldwide. Purpose of this study was to identify molecular markers that were significantly correlated with presence of glaucoma and outcome of glaucoma surgery. To accomplish this, we determined the profiles of pro-inflammatory cytokines in the aqueous humor of 101 glaucoma patients; 31 primary open angle glaucoma (POAG), 38 pseudoexfoliation glaucoma (PEG), and 32 neovascular glaucoma (NVG). We also studied 100 normal subjects as controls. In eyes with POAG or PEG, the level of interleukin (IL)-1α, IL-2, IL-4, IL-8, IL-23, and CCL2 were significantly elevated. In the NVG eyes, many inflammatory cytokines were also highly elevated. IL-8 had the highest odds ratio, and levels of IL-8 and CCL2 were significantly correlated with preoperative IOP or visual field defects in PEG eyes. Principal component analysis showed that IL-8 had the highest association to the IOP-cytokine component, and Cox proportional hazard model indicated that an elevation of IL-8 was a significant risk of filtering surgery failure. Together with modeling of their interactions and prognosis, IL-8 elevation is a significant risk factor both for detecting and managing glaucoma and may serve as a therapeutic target candidate to improve the prognosis of glaucoma surgery.

## Introduction

Glaucoma is a leading cause of blindness worldwide. Recent advances in diagnostic techniques have allowed clinicians to detect glaucoma effectively. Nevertheless, an early diagnosis and management is still difficult, and patients can develop significant visual field loss even while being treated with topical medications. Surgical interventions are required to reduce the elevated intraocular pressure (IOP) in eyes that are refractory to medications.

An elevation of IOP can be caused by a decrease in the outflow of aqueous humor through the trabecular meshwork, Schlemm’s canal, and the collector channels. Fibrotic changes or deposits of extracellular matrix into the trabecular meshwork can slow or block the outflow which then leads to an elevation of the IOP. Open angle glaucoma (OAG) often develops in eyes with the pseudoexfoliation syndrome which is associated with deposits of abnormal extracellular matrix in the meshwork tissue increasing the outflow resistance, and pseudoexfoliation glaucoma (PEG) often cause very high IOPs.

Neovascular glaucoma (NVG) can also cause very high IOPs, and the elevation is sustained. Neovascularization in the trabecular tissue and iris can cause severe inflammation which then blocks aqueous outflow and an elevation of the IOP. NVG typically develops as OAG with severe ocular ischemia due to central retinal vein occlusion or proliferative diabetic retinopathy.

Analyses of the transcriptome and proteome of glaucomatous eyes have shown that complex molecular events are associated with the development of glaucoma. It has been proposed that the aqueous humor contains molecular factors that are responsible for the slowing of aqueous outflow^1^.

In glaucomatous eyes refractory to medications, the good results of surgical interventions can fail after months or years which would then require a second surgery. One important cause of the failure is fibrotic changes at the surgical site or of the drainage bleb, and transforming growth factor (TGF)-β and chemokine (C-C motif) ligand 2 (CCL2), also referred to as monocyte chemoattractant protein 1 (MCP1), have been suggested to be the inflammatory mediators for the fibrotic changes^2^. Thus, it is reasonable to suggest that inflammatory mediators may cause or contribute to the reduced outflow and surgical failure. Although no unified molecular signature or prognosis determinant has been determined for OAG and NVG, a determination of the molecular mechanisms of such markers should contribute to the development of new interventions or therapeutic strategies to manage such eyes.

Thus, the purpose of this study was to determine the profiles of the pro-inflammatory cytokines in the aqueous humor of patients with primary open angle glaucoma (POAG), pseudoexfoliation glaucoma, and NVG. To accomplish this, statistical analyses were performed to determine which inflammatory cytokines were significantly correlated with the IOP and visual field defects. Analyses were also performed to identify cytokines that were significantly correlated with glaucoma filtering surgery failure, and their associations with prognosis were modelled using structural equation modeling. We shall show that there is a subset of inflammatory cytokines that is significantly associated with glaucomatous eyes, and the chemokine interleukin (IL)-8 may serve as a predictor of visual field alterations as well as surgical outcomes of trabeculectomy.

## Results

### Association of inflammatory cytokines with open angle glaucoma and neovascular glaucoma

One hundred and one eyes met the inclusion criteria; 31 eyes with POAG, 38 eyes with PEG, and 32 eyes with NVG. Of these, 83 eyes had undergone glaucoma surgery, viz., 46 eyes with trabeculectomy and 37 eyes with trabeculotomy. Four of these trabeculectomy eyes had a history of one uncomplicated glaucoma surgery more than 2 years and 9 months (ranging from 2 years 9 months to 8 years and 4 months) before the beginning of this study. The previous glaucoma surgery was trabeculectomy for 2 eyes and trabeculotomy for 2 eyes.

The mean age was 71.2 ± 2.0 years for the POAG patients, 73.8 ± 1.4 years for the PEG patients, and 66.5 ± 2.4 for the NVG patients. (Table 1) The preoperative IOP was 37.5 ± 2.5 mmHg for the NVG eyes which was significantly higher than that of the POAG group at 20.7 ± 0.8 mmHg and the PEG group at 24.0 ± 1.2 mmHg. Men made up 45.2% of the POAG eyes, 60.5% of the PEG eyes, and 59.4% for the NVG eyes.

**Table 1:** Patient Characteristics.

	Control	Primary open angle glaucoma	Pseudoexfoliation glaucoma	Neovascular glaucoma
Patients, *n*	100	31	38	32
Men, *n* (%)	49 (49.0)	14 (45.2)	23 (60.5)	19 (59.4)
Age	73.9 ± 1.0	71.2 ± 2.0	73.8 ± 1.4	66.5 ± 2.4
Preoperative IOP (mmHg)	12.1 ± 0.3	20.7 ± 0.8	24.0 ± 1.2	37.5 ± 2.5
Diabetes, *n* (%)	32 (32.0)	5 (16.1)	9 (23.7)	26 (81.3)

**Figure 1:**
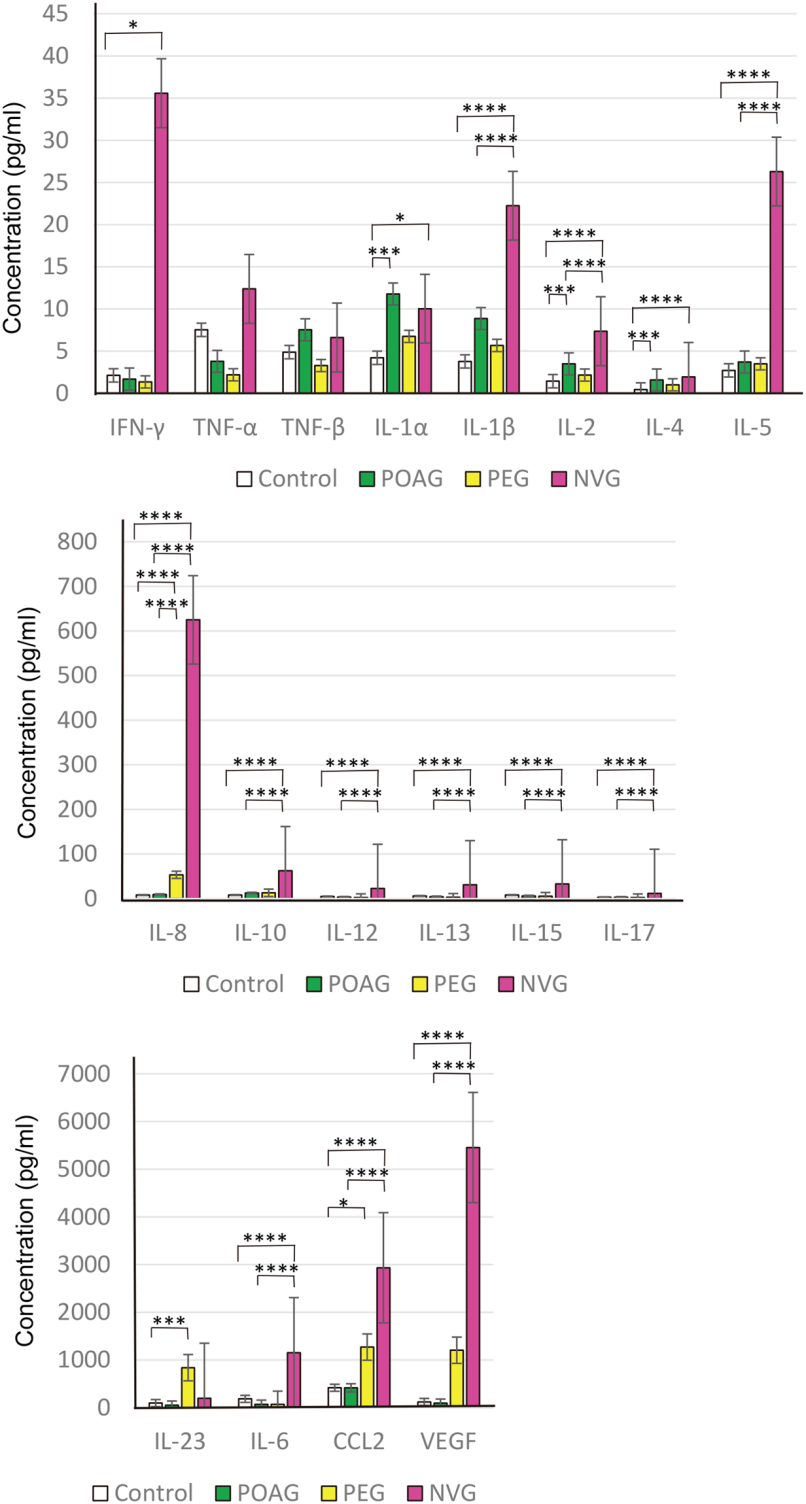
Elevation of inflammatory cytokines in the aqueous humor of eyes with primary open angle glaucoma (POAG), pseudoexfoliation glaucoma (PEG), and neovascular glaucoma (NVG). There is a significant elevation of IL-1α, IL-2, and IL-4 in the POAG eyes. In PEG eyes, levels of IL-8, IL-23, and CCL2 were significantly higher than in the normal control eyes. There is a significant elevation of IFN-γ, IL-1α, IL-1β, IL-2, IL-4, IL-5, IL-6, IL-8, IL-10, IL-12, IL-13, IL-15, IL-17, CCL2, and VEGF in the NVG eyes patients. ANOVA and post hoc tests. The concentrations are the means (pg/ml) and standard error of the means (SEM). Control: *n* = 100, POAG: *n* = 31, PEG: *n* = 38, NVG: *n* = 32 *P < 0.05; **P < 0.01; ***P < 0.005, ****P = 0.000.

To screen for markers of POAG, PEG, and NVG, we first determined the levels of cytokines in the aqueous fluid. Our results showed that the levels of IL-1α, IL-2, and IL-4, were significantly higher in the POAG eyes than in the normal control eyes (ANOVA, Fig. 1). In PEG eyes, the levels of IL-8, IL-23, and CCL2 were significantly higher than in the normal control eyes, and IL-8 was significantly higher than that in POAG eyes. The levels of IFN-γ, IL-1α, IL-1β, IL-2, IL-4, IL-5, IL-6, IL-8, IL-10, IL-12, IL-13, IL-15, IL-17, CCL2, and VEGF were significantly higher in the NVG eyes than in the normal control eyes, and IL-1β, IL-2, IL-5, IL-6, IL-8, IL-10, IL-12, IL-13, IL-15, IL-17, CCL2, and VEGF were higher than in the POAG eyes. The cytokines with the highest levels in the NVG eyes were VEGF, CCL2, IL-6, and IL-8, confirming the neovascular and inflammatory nature of NVG. All of these cytokines were significantly elevated with the degree of elevation dependent on the disease type, i.e., POAG, PEG, or NVG (*P* < 0.0005, Jonckheere Terpstra test). These results suggest disease-dependent glaucomatous changes of the eyes.

**Figure 2:**
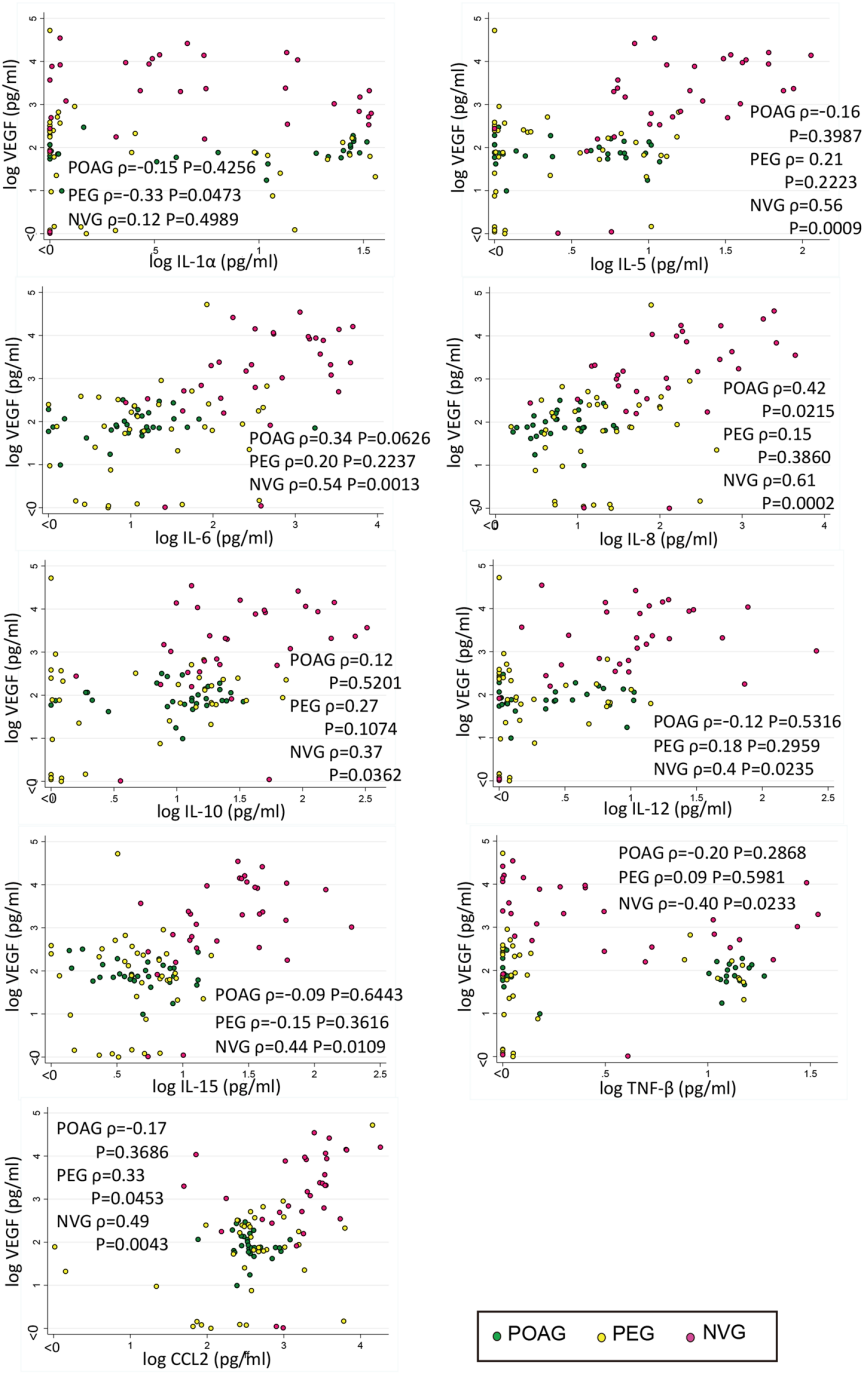
The levels of the cytokines that are significantly correlated with the VEGF levels in the aqueous humor of eyes with primary open angle glaucoma (POAG), pseudoexfoliation glaucoma (PEG), and neovascular glaucoma (NVG) are shown. In NVG eyes, IL-5, IL-6, IL-8, IL-10, IL-12, IL-15, and CCL2 are positively correlated with the VEGF levels. In POAG eyes, IL-8 is significantly correlated with the VEGF levels. In PEG eyes, CCL2 is positively correlated with the level of VEGF. Spearman correlation analysis. POAG: *n* = 30, PEG: *n* = 37, NVG: *n* = 32.

Vascular endothelial growth factor (VEGF) is a signature cytokine associated with NVG, and it plays significant roles in neovascularization, inflammation, and remodeling of the outflow system. Because the VEGF level probably represents the aggressiveness of the disease and refractoriness to treatments, we searched for cytokines that were significantly associated with the VEGF levels in the glaucomatous eyes. In the NVG eyes, the level of VEGF was positively correlated with the levels of IL-5, IL-6, IL-8, IL-10, IL-12, IL-15, and CCL2 (Spearman correlation analysis, correlation coefficient; ρ = 0.56 (*P* = 0.0009); ρ = 0.54 (*P* = 0.0013); ρ = 0.61 (*P* = 0.0002); ρ = 0.37 (*P* = 0.0362); ρ = 0.4 (*P* = 0.0235); ρ = 0.44 (*P* = 0.0109); and ρ = 0.49 (*P* = 0.0043)), respectively (Fig. 2). IL-8 had the highest correlation coefficient in the NVG eyes, and also had significant correlation in the POAG eyes (ρ = 0.42, *P* = 0.0215). In the PEG eyes, significant positive correlations were observed for CCL2 (ρ = 0.33, *P* = 0.0453). These findings suggest that these inflammatory cytokines, especially IL-8 and CCL2, may play a disease-dependent promoting role on the VEGF axis.

We then hypothesized that the inflammation-associated cytokines may also contribute to the characteristics of POAG, PEG, and NVG. To determine the consequences of the associations with these cytokines, we applied logistic regression analyses to evaluate associated risk of POAG, PEG, and NVG compared to normal control eyes. Disease-dependent significant associations were observed for IL-2, IL-4, IL-8, IL-10, and CCL2. Of these, IL-8 had the highest OR for eyes with PEG or NVG. (Table 2).

**Table 2:** Association of inflammatory cytokines with open angle glaucoma, pseudoexfoliation glaucoma, or neovascular glaucoma.

Cytokine (quintile)	POAG/PEG/NVG			POAG			PEG			NVG		
	Odds ratio	95% CI	*P*-value	Odds ratio	95% CI	P-value	Odds ratio	95% CI	P-value	Odds ratio	95% CI	P-value
IL-8	2.21	1.71–2.85	0.000	1.22	0.87–1.73	0.25	2.53	1.74–3.69	0.000	15.9	6.34–39.88	0.000
IL-2	1.79	1.43–2.25	0.000	1.72	1.24–2.39	0.001	1.29	0.96–1.72	0.09	4.34	2.57–7.30	0.000
IL-10	1.79	1.43–2.24	0.000	1.83	1.32–2.55	0.000	1.26	0.94–1.67	0.12	4.36	2.59–7.35	0.000
IL-4	1.67	1.35–2.07	0.000	1.92	1.40–2.64	0.000	1.46	1.12–1.92	0.006	1.78	1.31–2.43	0.000
CCL2	1.66	1.34–2.06	0.000	1.36	1.01–1.84	0.04	1.35	1.02–1.79	0.04	3.87	2.36–6.35	0.000

Logistic regression analyses (POAG/PEG/NVG, left) and multinomial logistic regression analyses (POAG, PEG, or NVG, right) after age adjustment, 95% confidence interval (CI). Primary open angle glaucoma (POAG), pseudoexfoliation glaucoma (PEG), and neovascular glaucoma (NVG). Odds ratio was calculated compared with normal control eyes as base outcome. Control: *n* = 100, POAG: *n* = 31, PEG: *n* = 38, NVG: *n* = 32.

### Elevation of inflammatory cytokines in glaucomatous eyes with visual field defects and IOP elevations

An IOP elevation is an important factor in the development of glaucomatous visual field defects and also in the refractoriness of the glaucomatous changes to treatment. The IOP levels were highest for the NVG eyes followed by the PEG eyes, and the POAG eyes had the lowest IOP levels (Table 1). Thus, we determined which inflammatory cytokines that were elevated in the aqueous humor of the glaucomatous eyes were significantly associated with the IOP elevation and the visual field defects.

**Figure 3:**
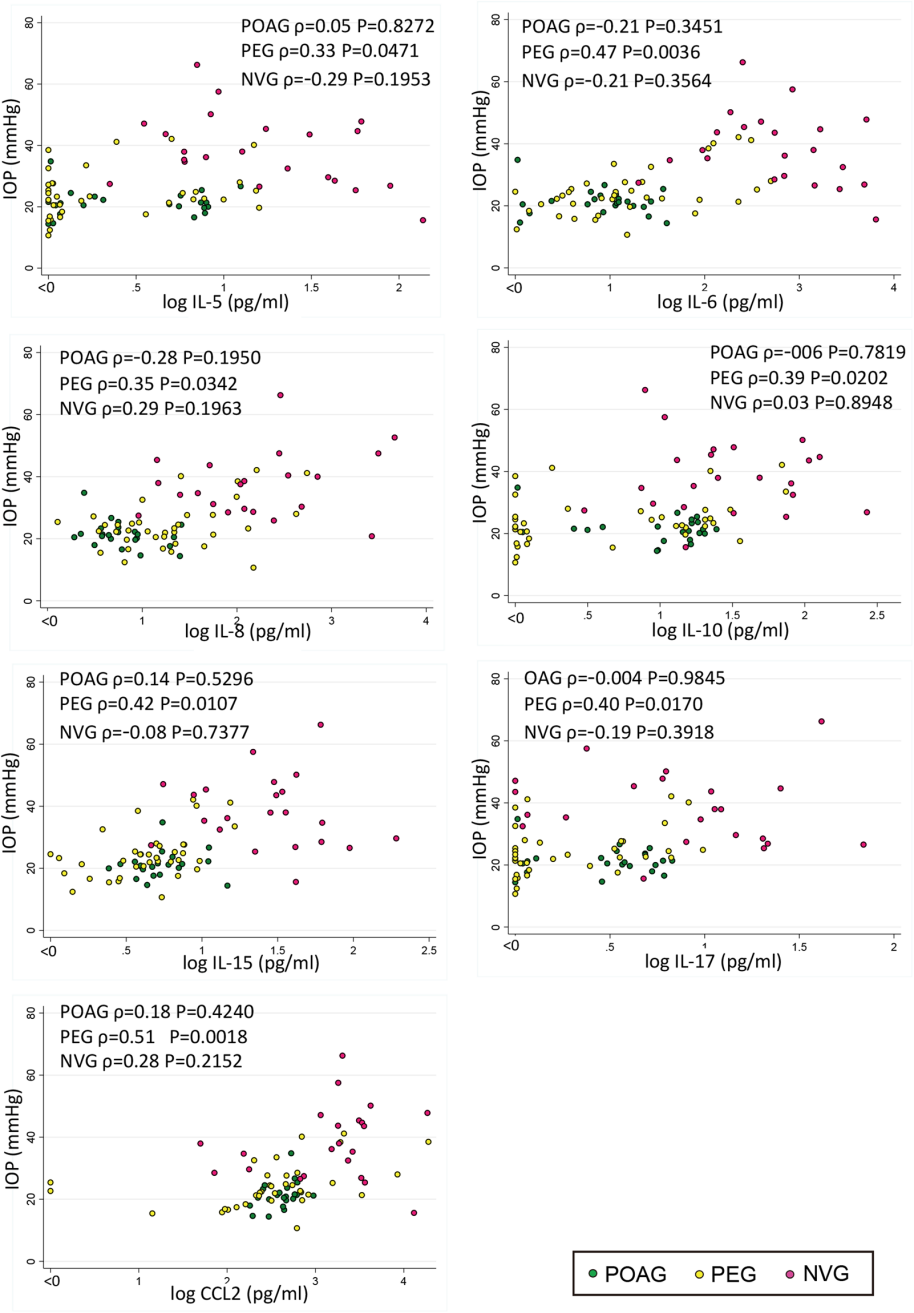
Cytokines that are significantly correlated with the intraocular pressure in eyes with pseudoexfoliation glaucoma (PEG) are shown. The levels of IL-5, IL-6, IL-8, IL-10, IL-15, IL-17, and CCL2 are significantly correlated with the IOP in eyes with PEG eyes. In eyes with primary open angle glaucoma or neovascular glaucoma, significantly correlated cytokines are not present. Spearman correlation analysis. POAG: *n* = 23, PEG: *n* = 36, NVG: *n* = 22.

Our results showed that IL-5, IL-6, IL-8, IL-10, IL-15, IL-17, and CCL2 were significantly correlated with the IOP levels in PEG eyes (Spearman correlation analysis, Fig. 3, IL-5 (ρ = 0.33, *P* = 0.0471), IL-6 (ρ = 0.47, *P* = 0.0036), IL-8 (ρ = 0.35, *P* = 0.0342), IL-10 (ρ = 0.39, *P* = 0.0202), IL-15 (ρ = 0.42, *P* = 0.0107), IL-17 (ρ = 0.40, *P* = 0.017), and CCL2 (ρ = 0.51, *P* = 0.0018)).

**Figure 4:**
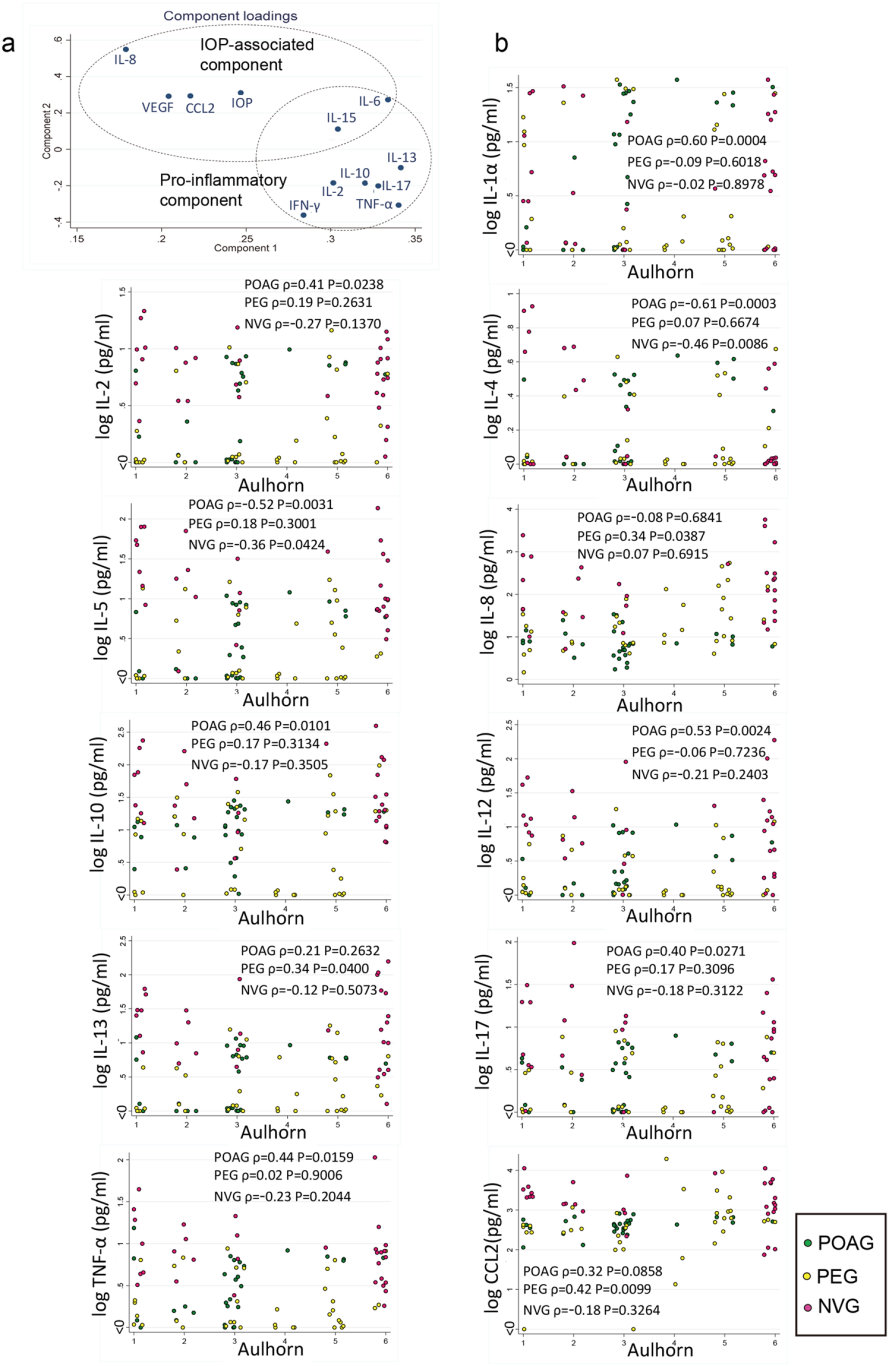
Association of cytokines with preoperative intraocular pressure (IOP) and visual field defects. (**a**) Results of principal component analysis of cytokines (quintile) associated with preoperative IOP levels. Pro-inflammatory component 1 is mainly composed of IL-13, TNF-α, IL-6, IL-17, IL-10, IL-15, IL-2, and IFN-γ. The IOP-related component is composed of IL-8, IL-15, VEGF, CCL2, and IL-6. IL-8 has the highest loading. *n* = 77. (**b**) In the pseudoexfoliation glaucoma eyes, IL-8, IL-13, and CCL2 have a significant positive correlation with the visual field defects. In primary open angle glaucoma eyes, IL-1α, IL-2, IL-10, IL-12, IL-17, and TNF-α are positively correlated with the visual field defects. Spearman correlation analysis. POAG: *n* = 30, PEG: *n* = 37, NVG: *n* = 32.

To further analyze the IOP-related cytokines, a principal component analysis was performed on these cytokines. The results showed that the primary component was a pro-inflammatory one, and the secondary component was an IOP-associated one which comprised IL-6, IL-8, IL-15, VEGF, and CCL2 (Fig. 4a). Of these, IL-8 had the highest loading to the IOP associated component.

Another important aspect of eyes with glaucoma is the presence of glaucomatous optic neuropathy which is manifested by the presence of visual field defects. Therefore, we next examined which of the cytokines were significantly associated with the visual field defects in glaucoma patients.

Among the cytokines, IL-8 and IL-15 (quintile) had the highest significant trends with the stage (Aulhorn classification) of the visual field defect (*P* < 0.05, Jonckheere-Terpstra trend test). In the PEG eyes, IL-8, IL-13, and CCL2 had positive correlations with the visual field defects (Fig. 4b, IL-8 (ρ = 0.34, *P* = 0.0387), IL-13 (ρ = 0.34, *P* = 0.04), and CCL2 (ρ = 0.42, *P* = 0.0099)). In POAG eyes, IL-1α, IL-2, IL-10, IL-12, IL-17, and TNF-α were positively correlated with the visual field (IL-1α (ρ = 0.60, *P* = 0.0004), IL-2 (ρ = 0.41, *P* = 0.0238), IL-10 (ρ = 0.46, *P* = 0.0101), IL-12 (ρ = 0.53, *P* = 0.0024), IL-17 (ρ = 0.40, *P* = 0.0271), and TNF-α (ρ = 0.44, *P* = 0.0159)).

These findings indicate that IL-8 was significantly correlated with the IOP and the visual field defects in eyes with PEG. Therefore, we next calculated the partial correlation of IL-8 and visual field defects under the assumption that the level of IOP affected the visual field defects in PEG eyes. IL-8 (quintile) was significantly correlated with the visual field defects under this condition (partial correlation coefficient: 0.34, *P* = 0.01).

### Association of refractoriness to surgical intervention with elevated IL-8

We next examined how these inflammatory cytokines were associated with the surgical outcomes focusing on filtering surgery. The Cox proportional hazard model was used to determine the association of the preoperative cytokine levels with the postoperative outcome. Significant associations with a poor outcome were observed only for the levels of IL-8 (Table 3). The elevated IL-8 level had a significant hazard ratios (HR) in all the three types of glaucoma, and PEG eyes had a HR of 55.4 (per quintile, *P* = 0.000) indicating that the elevation of IL-8 was associated with surgical failure. The preoperative IOP level was not associated with surgical failure (data not shown).

**Table 3:** Association of IL-8 with poor surgical outcome of trabeculectomy using Cox proportional hazard model.

Effect on surgical outcome	Primary open angle glaucoma			Pseudoexfoliation glaucoma			Neovascular glaucoma		
	HR	95% CI	P-value	HR	95% CI	P-value	HR	95% CI	P-value
IL-8 (quintile)	14.0	2.34–83.3	0.004	55.4	11.2–272.8	0.000	5.22	1.25–21.9	0.02
VEGF (quintile)	0.30	0.17–0.53	0.000	0.11	0.05–0.23	0.000	0.28	0.12–0.67	0.004
CCL2 (quintile)	—	—	—	0.51	0.32–0.80	0.003	—	—	—
age	—	—	—	1.41	1.27–1.57	0.000	1.15	0.91–1.47	0.24

*n* = 46, Primary open angle glaucoma *n* = 8, pseudoexfoliation glaucoma *n* = 17, neovascular glaucoma *n* = 21. Hazard ratio (HR), 95% confidence interval (CI).

**Figure 5:**
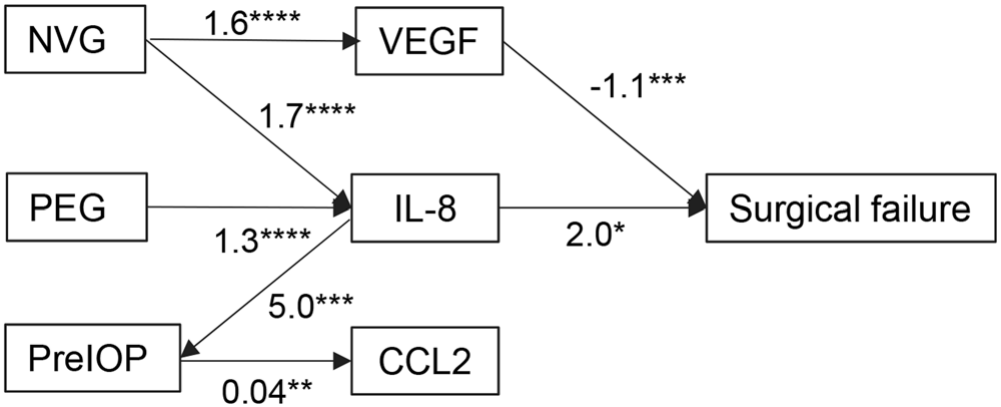
Association of elevated IL-8 and prognosis. Association of cytokines, surgical failure, and preoperative IOP in eyes that had undergone trabeculectomy are shown in the path diagram of exponential survival analysis using Generalized Structural Equation Modeling (GSEM). IL-8 (quintile) and VEGF (quintile) are significantly associated with surgical failure (shown as paths). Preoperative IOP (preIOP) is significantly associated with IL-8 and CCL2 (quintile). The disease type, e.g., pseudoexfoliation glaucoma (PEG), neovascular glaucoma (NVG), coded as 0/1, is significantly associated with the levels of VEGF and IL-8. Numbers on path indicates significant coefficients in GSEM analysis. For surgical failure, numbers indicate significant hazard ratio (HR) of IL-8 and VEGF. *n* = 42 *P < 0.05; **P < 0.01; ***P < 0.005; ****P < 0.000.

The generalized structural equation modeling (GSEM) analysis using exponential survival model was used to determine how the IOP-related cytokines were associated with the IOP and failure of trabeculectomy directly or indirectly (Fig. 5). The selection of the path connecting the cytokines and IOP was determined using fitting indices, Akaike information criteria (AIC), and Bayesian information criteria (BIC)^3^. When the surgical failure was assessed for association with the level of the preoperative cytokines and IOP levels, an elevation of IL-8 was significantly associated with surgical failure. This association was not associated with the preoperative IOP because the path from the preoperative IOP to surgical failure was not significant (path not shown). Consistent with disease type-dependent cytokine elevations (Fig. 1), eyes with PEG or NVG showed significant associations with the cytokine levels. When we assessed the association of the IOP with the cytokine levels, IL-8 and CCL2 had significant associations with the IOP elevation.

## Discussion

Pathological changes, such as inflammation, ischemia, hypoxia, oxidative stress, endoplasmic reticulum (ER) stress, tissue remodeling, and fibrotic changes, in the trabecular outflow system have been reported to be associated with an elevation of the IOP. We focused on the inflammatory aspects of glaucomatous eyes and analyzed the inflammatory aspects of POAG, PEG, and refractory NVG. Our results showed that the level of IL-8 in the aqueous might be a potential candidate molecule that can predict the clinical outcome of surgical interventions in eyes with refractory glaucoma.

Earlier analyses of the aqueous humor indicated that there were significant elevations of inflammatory cytokines for different types of glaucomatous diseases in different settings. For POAG, IL-6, IL-8, IL-12, IFN-γ, TNF-α, and CXCL9 were found to be significantly elevated^4–7^. For PEG, significant elevations of IL-4, IL-6, IL-8, IL-16, CCL13, CCL15, CCL22, CCL24, CXCL13, and CXCL16 were found^8,9^. For POAG and PEG, CCL2, IL-8, TGF-β1, serum amyloid A (SAA), and TNF-α were found to be significantly elevated^10,11^. The findings in all of these studies indicated an association of glaucoma with IL-8 which we found to be a critical risk factor.

The level of the cytokines in the aqueous humor of eyes with the inflammation types of glaucoma, e.g., secondary glaucoma by uveitis, has also been examined. In uveitic glaucoma, IL-6, IL-8, CCL2, TNF-α, and VEGF were found to be elevated in the aqueous^12^. The Posner-Schlossman syndrome is a disease characterized by an acute elevation of the IOP with aqueous humor inflammation. A viral infection is suspected to be one cause because viral genomes have been detected in the aqueous samples. Li *et al*. reported that eyes with Posner-Schlossman syndrome had a significant elevation of IL-8, CCL2, CCL4, GCSF, and TGF-β irrespective of whether there is a CMV infection^13^. Thus, these cytokine profiles of glaucoma associated with inflammation appear similar to that in eyes with OAG, PEG, and NVG^13^.

NVG is a type of secondary glaucoma that is most refractory to medical and surgical treatments. NVG typically develops after a central retinal vein occlusion or in eyes with proliferative diabetic retinopathy when the tissues of the affected eyes develop from severe ischemia. Because an important cause of glaucoma is an ischemic condition, NVG can be considered one arm of glaucoma. Moreover, NVG has proinflammatory conditions as well as outflow resistance due to abnormal new vessels in the angle. As is found after a breakdown of the blood:aqueous barrier, a wide spectrum of inflammatory cytokines including VEGF are highly elevated resembling a cytokine storm (Fig. 1). This cytokine profile is consistent with a previous report showing severe elevations of IL-6, IL-8, CCL2, TNF-α, and PDGF in eyes with NVG^14^.

Because the IOP is an important target for glaucoma treatments, we determined the association of the cytokine levels, elevation of the IOP, and optic disc damage. Previously, significant correlations were found for TGF-β, IL-8, and SAA in eyes with OAG and PEG^10^, and for CXCL13, CCL8, and CCL3 in PEG^8^, and for IL-8, IP-10, CCL2, and CCL4 in POAG and NVG^15^. We noted that a number of cytokines were associated with the elevation of the IOP especially in PEG eyes. For example, the levels of IL-5, IL-6, IL-8, IL-10, IL-15, IL-17, and CCL2 were significantly correlated with the IOP. This indicated that there was a broader influence of the intracameral cytokines on the IOP or IOP on cytokines in glaucomatous eyes. However, the correlations of the level of cytokines and IOP may reflect indirect associations (Fig. 5).

Based on previous analyses of glaucomatous eyes of different etiologies with or without inflammation, CCL2 and IL-8 appears to be consistently elevated in all. Thus, these cytokines may also be involved in the pathology of glaucoma or reflect the elevation of the IOP. However, it is difficult to understand how such cytokines are involved in the glaucomatous pathology. In our study, we determined the model using time direction of path to surgical failure based on how the model fits the observational data, and the resultant model was highly significant. However, we cannot conclude on a causal relationship considering the possibility of covariates that have affected the relationship. Proof of causality generally requires randomized control studies that can be performed in the future when ethical issues are resolved.

We analyzed the complex interactions of the IOP and cytokines using principal component analysis. There were two independent components; a pro-inflammatory component and an IOP-associated component (Fig. 4a). The major association of the IOP without an inflammatory component was with IL-8, CCL2, and VEGF. These associations are reasonable because all of these cytokines are known to affect the outflow facility, endothelial permeability, remodeling of extracellular matrix, and recruitment of bone marrow-derived fibrotic cells.

The significant associations of the level of these cytokines with the IOP suggest that they may also have significant associations with the glaucomatous visual field defects. Huang *et al*. analyzed eyes with POAG and mean visual field defect (MD) of <12 and MD of ≥12, and they reported a tendency of higher serum IL-4 and IL-6 levels to be associated with more advanced visual field defects^16^. However, in aqueous humor, IL-4 was negatively correlated with visual field defects in POAG and NVG eyes (Fig. 4b). Kuchtey *et al*. reported that eyes with more advanced visual field defects had higher IL-8 levels in the aqueous humor than eyes with less advanced visual field defects^6^. We confirmed that there are significant associations of the presence of visual field defects and higher levels of IL-8 in PEG eyes. This may suggest that PEG eyes tend to have higher and longer duration of IOP elevations. However, whether this is an indirect bystander effect or somehow contributed to a visual field defect needs experimental validations or clinical trial studies.

The prognosis for OAG is dependent on the outcome of surgical interventions. Following glaucoma filtering surgery, fibrotic changes of the bleb is a risk factor for surgical failure. Cytokines that affect the filtering surgery were analyzed by assessments of the aqueous humor samples. In OAG patients with a hypertensive bleb after a Molteno implant surgery (>38 mmHg), the levels of IL-6, IL-8, IL-10, chemokine (C-X-C motif) ligand 1 (CXCL1), CCL2, and TGF-β2 were significantly elevated in the aqueous humor^2^. Because the hypertensive phase reflects a transient IOP rise after surgery, the cytokine elevation appears to reflect the IOP rise. When OAG patients undergoing trabeculectomy were analyzed, patients with high CCL2 had a lower probability of success by survival analysis^17^. Our GSEM model (Fig. 5b) that focused on the molecular pathology of eyes undergoing trabeculectomy indicated that an elevation of IL-8 reflected high IOP levels which induces an elevation of CCL2. Thus, the IL-8 elevation may be a true marker for surgical failure.

Several mechanisms have been postulated on how the IOP level is affected, and how an elevated IOP can damage the outflow facility. These mechanisms include fibrotic changes or remodeling of the outflow system, and the loss or dysfunction of the trabecular meshwork by pro-inflammatory cytokines belonging to the IOP-associated components (Fig. 4a). Of these, IL-8 is known to recruit macrophages, and to also have profibrotic properties^18^. IL-8 was recently shown to be related to the fibrotic pathology in patients with idiopathic pulmonary fibrosis^18^. Thus, IL-8 not only stimulates macrophage recruitment but also promotes the proliferation of fibrogenic mesenchymal progenitor cells leading to the generation of fibrotic lesions.

CCL2 is known to be involved in tissue remodeling. CCL2 induces the synthesis of type 1 collagen in fibroblasts by the production of TGF-β^19^. CCL2 also induces the formation of matrix metalloproteinase-1 and tissue inhibitor of metalloproteinase-1 (TIMP-1). IL-6 is profibrotic and induces TGF-β and pseudoexfoliation material, fibrillin-1, and LTB-1 which will disrupt the outflow facility^9^. VEGF is also well known to increase the vascularity and to play a role as a fibrotic mediator^20^. All of these changes lead to the development of fibrotic changes and tissue remodeling.

There are some limitations for this study. Patients enrollment was based mainly on referral to the Tottori University Hospital as a tertiary referral hospital, and this may have caused an enrollment of more refractory cases. Because of nature of this population and prospective design of this study, we did not exclude small cases with previous history of glaucoma surgery of more than 2 years which had silent conjunctiva, i.e., no bleb formation. In addition, the effect of previous surgery was assumed to have not influenced the results. These characteristics of the population may have caused over or under-estimation of effects of each cytokine. Moreover, the calculated roles of each cytokines are based on statistical association and may not reflect causality.

To summarize, IL-8 and a subset of inflammatory cytokines are part of the IOP component in glaucomatous pathology, and they probably act through a complex interplay of the pro-inflammation in an inflammatory milieu. Of these, IL-8 contributes significantly to this component. Elevations in the level of IL-8 can serve as a prognostic marker for surgical failure, and its modulation may form the basis of new therapies.

## Methods

### Eligibility criteria and diagnosis

The study protocol was approved by the Ethics Committee of the Tottori University, and the procedures used conformed to the tenets of the Declaration of Helsinki. An informed consent was obtained from all of the participants after an explanation of the protocol.

POAG was diagnosed by the presence of an open iridocorneal angle, glaucomatous cupping of the optic disc, and visual field defects (Humphrey Visual Field Analyzer, Carl Zeiss, Dublin, CA) without signs of secondary glaucoma. The IOP was measured by Goldman applanation tonometry. PEG was diagnosed by the presence of an open angle, glaucomatous cupping of the optic disc, and visual field defects with the presence of deposits of pseudoexfoliation material. A diagnosis of neovascular glaucoma was made by the presence of neovascularization in the anterior chamber angle or iris with ischemic lesions in the fundus determined by fluorescein angiography (FA).

This study was designed as a prospective, open cohort study. The inclusion criteria were patients with POAG, PEG, or NVG that had undergone surgical interventions including glaucoma surgery, cataract surgery, or intravitreal aflibercept injections for NVG. Each subject had signed a written consent form to study their medical records before the surgical interventions from September 2008 to November 2016.

Eyes that had undergone any other intraocular surgery within 3 months of the beginning this study or eyes with any functioning filtering bleb were excluded. Eyes that had active inflammation due to previous surgeries, including more than one glaucoma surgery, were also excluded. Four eyes had history of one uncomplicated glaucoma surgery more than 2 years and 9 months before.

The indication for glaucoma surgery was an inadequately controlled IOP (>20 mmHg) by maximal topical medications. For NVG eyes, all the patients underwent trabeculectomy which was preceded by an intravitreal injection of aflibercept.

For the analyses of all the subjects, the aqueous humor from normal conventional cataract surgery patients without glaucoma served as controls.

On the day of the surgery or intravitreal injection, 100–200 μl of aqueous humor was collected by paracentesis before the surgery or injection. The aqueous samples were frozen until used for the cytokine measurements. For controls, aqueous samples were collected from 100 normal subjects prior to undergoing routine cataract extraction.

### Follow-up and criteria for surgical failure

The IOP measured at last visit before collection of aqueous humor was used as preoperative IOP. To determine the effects of the preoperative level of cytokines on the postoperative IOP, the IOP of the 79 eyes that had undergone glaucoma surgery (59 OAG; 20 NVG) were measured monthly and assessed for surgical failure using Cox proportional hazard model and Generalized Structural Equation Modeling (GSEM). The criterion of failure for all these analyses was defined as the need of a second surgery due to an elevated IOP (>20 mmHg) with maximum topical glaucoma medications.

The risk of high levels of preoperative cytokines, preoperative IOP, and clinical characteristics affecting the success of the surgery was determined by Cox proportional hazard model and GSEM analysis. The hazard ratio of cytokines was calculated after adjustments for the clinical parameters. The visual field defects determined at the last visit before the collection of the aqueous humor were analyzed for their correlations with the aqueous cytokine levels.

### Measurement of cytokines in aqueous humor

Aqueous humor samples were analyzed for the levels of IL-1α, IL-1β, IL-2, IL-4, IL-5, IL-6, IL-8, IL-10, IL-12, IL-13, IL-15, IL-17, IL-23, IFN-γ, TNF-α, TNF-β, VEGF, and CCL2 as described in detail^21–23^. The concentrations of these cytokines were measured with commercially available ELISA kits: Quansys Biosciences (West Logan, UT) for IL-1α, IL-1β, IL-2, IL-4, IL-5, IL-6, IL-8, IL-10, IL-12, IL-13, IL-15, IL-17, IL-23, IFN-γ, TNF-α, and TNF-β; PeproTech kit (Rocky Hill, NJ) for CCL2; and R&D Systems kit (Minneapolis, MN) for VEGF.

Briefly, the aqueous humor samples were diluted four-fold using the diluent provided by the kits and processed according to the manufacturers’ instructions. The diluted samples were incubated on the capture antibody-coated plates overnight at 4 °C and processed for detection by chemiluminescence.

### Statistical analyses

Data are presented as the means ± standard error of the means. Logistic regression analyses were performed to compute the odds ratios (OR) based on quintiles of each cytokine level. Each cytokine quintile was compared with the lowest quintile as the reference category^22,23^. Unpaired *t* tests, Mann-Whitney U tests, ANOVA and post hoc tests were used to determine whether the differences between groups were statistically significant. Jonckheere-Terpstra trend tests were used to determine the ordered differences among the groups. Generalized structural equation modeling (GSEM) analysis using exponential survival model was performed to calculate the relationship of cytokines levels, IOP, and hazard ratio of surgical failure. For logistic regression analysis and GSEM, eyes with PEG, NVG, and their clinical characteristics were coded as present or absent (0/1). The model was selected based on its fitting to the data using Akaike information criteria (AIC) and Bayesian information criteria (BIC)^3^. Statistical analyses were performed with Stata 15 software (College Station, TX). A *P* < 0.05 was taken to be significant.

## Data Availability

The datasets generated during and/or analyzed during the current study are available from the corresponding author on reasonable request.
